# Body-as-Subject in the Four-Hand Illusion

**DOI:** 10.3389/fpsyg.2018.01710

**Published:** 2018-09-19

**Authors:** Caleb Liang, Yen-Tung Lee, Wen-Yeo Chen, Hsu-Chia Huang

**Affiliations:** ^1^Department of Philosophy, National Taiwan University, Taipei, Taiwan; ^2^Graduate Institute of Brain and Mind Sciences, National Taiwan University, Taipei, Taiwan

**Keywords:** body-as-subject, body-as-object, four-hand illusion, first-person perspective, body ownership

## Abstract

In a recent study (Chen et al., 2018), we conducted a series of experiments that induced the “four-hand illusion”: using a head-mounted display (HMD), the participant adopted the experimenter's first-person perspective (1PP) as if it was his/her own 1PP. The participant saw four hands via the HMD: the experimenter's two hands from the adopted 1PP and the subject's own two hands from the adopted third-person perspective (3PP). In the active four-hand condition, the participant tapped his/her index fingers, imitated by the experimenter. Once all four hands acted synchronously and received synchronous tactile stimulations at the same time, many participants felt as if they owned two more hands. In this paper, we argue that there is a philosophical implication of this novel illusion. According to Merleau-Ponty (1945/1962) and Legrand (2010), one can experience one's own body or body-part either as-object or as-subject but cannot experience it as both simultaneously, i.e., these two experiences are mutually exclusive. Call this view the Experiential Exclusion Thesis. We contend that a key component of the four-hand illusion—the subjective experience of the 1PP-hands that involved both “kinesthetic sense of movement” and “visual sense of movement” (the movement that the participant sees via the HMD)—provides an important counter-example against this thesis. We argue that it is possible for a healthy subject to experience the same body-part both as-subject and as-object simultaneously. Our goal is not to annihilate the distinction between body-as-object and body-as-subject, but to show that it is not as rigid as suggested by the phenomenologists.

The target of this paper is a philosophical view about the phenomenological distinction between “body-as-object” and “body-as-subject” (Merleau-Ponty, 1945/1962; Legrand, 2010). According to this view, one can experience one's own body or body-part either *as-object* or *as-subject* but *cannot* experience it *as both at the same time*. Merleau-Ponty once gave a nice illustration:

“I can, with my left hand, feel my right hand as it touches an object, the right hand as an object is not the right hand as it touches: the first is a system of bones, muscles, and flesh brought down at a point of space, the second shoots through space like a rocket to reveal the external object in its place. In so far as it sees or touches the world, my body can therefore be neither seen nor touched” (1945/1962, 105).

The idea is that when the right hand is touched, it is experienced *as-object*, i.e., as a system of bones and muscles in a particular location that can be seen or touched. This is an experience of body-as-object. When the same right hand touches something, it is experienced *as-subject* that performs an active movement. Or, in the case of a whole body, the body is experienced as a bodily subject that perceives and acts in the world.

Two quick remarks regarding the notion of “subject” in this paper: first, a subject of conscious state is one that possesses the first-person perspective (1PP) on that state. In this regard, we agree with Legrand when she says that a 1PP “is tied to a self in the sense of being tied to the point of view of the experiencing, perceiving, acting subject” (2007a, p. 584). Second, we also agree with Legrand (2006, 2010) that a subject of conscious state is not a Cartesian ego; rather, it is essentially a bodily self. On this view, the “body” in “body-as-subject” is construed as an “experiencing body” and “bodily agent.” Hence, the experience of body-as-subject is an experience at the person-level^1^.

Merleau-Ponty emphasized that an experience of body-as-subject is fundamentally different from experiencing the same body as-object: “I observe external objects with my body, I handle them, examine them, walk round them, but my body itself is a thing which I do not observe: in order to be able to do so, I should need the use of a second body which itself would be unobservable” (1945/1962, p. 104). This suggests that I can experience my body as-subject only when it is *not* experienced as-object. These two types of experiences cannot take place in the same body or body-part *simultaneously*, that is, they are mutually exclusive.

To articulate this view, consider what Merleau-Ponty called “double sensations”: “When I press my two hands together, it is not a matter of two sensations felt together as one perceives two objects placed side by side, but of an ambiguous set-up in which both hands can alternate the roles of ‘touching' and being ‘touched.' What was meant by talking about ‘double sensations' is that, in passing from one role to the other, I can identify the hand touched as the same one which will in a moment be touching” (1945/1962, p. 106). In this case, both of my hands are pressing each other and being pressed at the same time. The idea is that, even so, the experiences of touching and being touched are incompatible such that they alternate between the two hands. In Merleau-Ponty's own words, “the two hands are never simultaneously in the relationship of touched and touching to each other” (1945/1962, p. 106). Taking this idea into account, Merleau-Ponty's view can be stated as follows:

“Body-as-object and body-as-subject are two different and incompatible modes of experience, such that they cannot take place simultaneously in the same body or body-part; rather, there can only be an alteration between the two modes of experience.”

We will call this view the *Experiential Exclusion Thesis*.

Regarding body-as-subject, Merleau-Ponty further said that “What prevents its ever being an object … is that it is that by which there are objects. It is neither tangible nor visible in so far as it is that which sees and touches. The body therefore is not one more among external objects … the body no longer conceived as an object of the world, but as our means of communication with it” (1945/1962, p. 105, 106). In another place, Merleau-Ponty said that “I move external objects with the aid of my body, which takes hold of them in one place and shifts them to another. But my body itself I move directly, I do not find it at one point of objective space and transfer it to another, I have no need to look for it, it is already with me” (1945/1962, p. 108). In these passages, Merleau-Ponty explained how the experiences of one's body and body part differ from the experiences of external objects. He thinks that the body that perceives (or the hand that touches) is not experienced as an object because it is the means via which we interact with the environment. The body is, so to speak, the channel through which external objects may be present to us. Hence, in so far as we perceive external objects through the body, the body itself is not the object of perception^2^. These considerations can be taken to support the Experiential Exclusion Thesis.

This distinction between “body-as-object” and “body-as-subject” has been developed and defended by several philosophers who have done interdisciplinary works in-between phenomenology and cognitive science (Gallagher, 2005, 2012; Zahavi, 2005, 2014; Legrand, 2006, 2007a,b, 2010). According to Legrand, the case of body-as-object is where one's body “is taken *as-intentional-object*, i.e., as the object toward which one's intentional act of consciousness is directed” (Legrand, 2010, p. 187). For example, when I look at my body in a mirror, I experience my body as the object of vision. Contrarily, in the case of body-as-subject, one precisely does *not* experience the body as any sort of intentional object. To use the same example: although my attention is focused on the image of the body in the mirror, I still implicitly experience myself as the one *who* is looking at the mirror. This experience of self as the perceiving subject illustrates the sense of body-as-subject (Legrand, 2010, p. 188). Following Merleau-Ponty (1945/1962), Legrand holds that body-as-object and body-as-subject are fundamentally different and incompatible modes of experience: “By definition, the body-*as-subject* is itself *absent*-as-intentional-object” (Legrand, 2010, p. 189, our emphasis, cf. also 193)^3^. The same body or body-part cannot be in both modes simultaneously; it can only be either one or the other.

In this paper, we challenge the above view of Merleau-Ponty and Legrand. We recently designed a series of experiments that induced the “four-hand illusion” such that the participants felt as if they possessed an additional pair of hands (Chen et al., 2018)^4^. After describing the experiments, we show that a key component of the four-hand illusion rules out the “alternation account” of bodily experience. It presents a strong case that experiences of body-as-object and body-as-subject are not always incompatible with each other, and hence provides an important counter-example against the Experiential Exclusion Thesis. We argue that it is possible for a healthy subject to experience the same body-parts both as-subject and as-object simultaneously. Then a few remarks will be made regarding other potential counter-examples. Our goal is not to annihilate the distinction between body-as-object and body-as-subject, but to show that it is not as rigid as suggested by the phenomenologists^5^.

## The four-hand illusion

**Figure 1:**
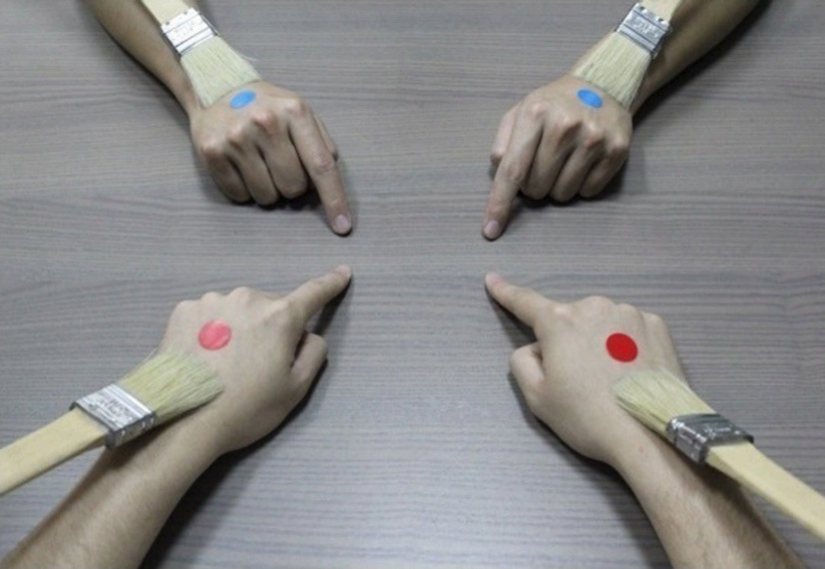
The set-up of the four-hand illusion. In Experiments 3, the experimenter's hands were seen via the HMD from the adopted 1PP with red tags, and the participant's own hands were seen via the HMD from the adopted 3PP (180° reverse) with blue tags. Both the participant and the experimenter tapped their index fingers and received tactile stimulations. To measure SCR, two single-use foam electrodes were attached to the inner side of the participant's left palm. The wires were carefully put under the participant's arm. So both the electrodes and the wires would not be seen by the participant via the HMD. This figure and its descriptions are adopted from Chen et al. (2018).

The experimental set-ups combined the 1PP and the third-person perspective (3PP). The participant wore a head-mounted display (HMD) connected with a stereo camera positioned right beside the experimenter. Sitting face to face, both the participant and the experimenter placed their hands on a table. After the participant put on the HMD, a color tag was attached to the back of all four hands^6^. Experiment 1 was the passive four-hand condition; Experiments 2 was the active four-hand condition without tactile stimulations. For our purpose, the key was Experiment 3—the active four-hand condition with tactile stimulations (Figure 1)^7^. Through the HMD, the participant adopted the experimenter's visual 1PP as if it was his/her own 1PP. The participant saw via the HMD an image of four hands: the experimenter's hands (with red tags) were seen from the adopted 1PP such that they appeared as if the participant was directly looking down at his/her own hands (hereafter, the 1PP-hands), and the participant's own hands (with blue tags) from the adopted 3PP in the opposite direction (180°) (hereafter, the 3PP-hands). All four hands were brushed synchronously for 60 s. Then both the participant and the experimenter were asked to tap on the table with their index fingers about once per second. After about 15 s, the participant was instructed to stop tapping, followed by a break for 10 s, and then the same tapping cycle was repeated. During these two tapping cycles, including the break in between, all four hands continued to be brushed synchronously. In the synchronous condition, the experimenter tapped his index fingers synchronously with respect to the participant's, such that the participant saw all four hands acting in exactly the same pattern. In the asynchronous condition, the experimenter intentionally tapped his index fingers asynchronously with respect to the participant's finger movements with time differences ranging from about 0.4–0.6 s. Each condition was followed by skin conductance reflectance (SCR) measurements on the experimenter's and the subject's hands as well as the questionnaire presented on the HMD. The whole procedure took about 180 s.

The distinctive feature of Experiment 3 was that it involved both “kinesthetic sense of movement” (the subject feels his/her own active movement via kinesthesis and proprioception) and “visual sense of movement” (the movement that the participant sees via the HMD), which were integrated in the synchronous condition. Here are the main experimental results: compared with Experiments 1 and 2, we found that the four-hand illusion was induced in the synchronous condition of Experiment 3. The averages of the key questionnaire statement (Q7, Table 1) were significantly higher in the synchronous condition than in the asynchronous condition. Sixty-eight percent of the participants in the synchronous condition answered positively that they had two *more hands*^8^. This was supported by various analyses^9^. First, different statistical comparisons showed that both the sense of ownership of the 1PP-hands and the sense of ownership of the 3PP-hands were induced in this condition. Second, compared with Experiments 1 and 2, the sense of body ownership and the sense of agency of the 1PP-hands were significantly stronger in Experiment 3. Together these results suggest that the four-hand illusion was successfully induced in Experiment 3. The synchronous finger tapping and synchronous tactile stimulations jointly contributed to inducing the four-hand illusion.

**Table 1:** Questionnaire statements.

**Category**	**Questionnaire statements**
Body ownership	1. It felt as if the hands with red tags were mine
	2. It felt as if the hands with blue tags were mine
Subjective tactile location	3. The touches that I felt were located on the hands with red tags
	4. The touches that I felt were located on the hands with blue tags
Agency	5. It felt as if I could control the hands with red tags
	6. It felt as if I could control the hands with blue tags
Key illusion	7. At a certain point, it felt as if I had two more hands
Control question	8. I felt that my hands were brushed

## Challenging the experiential exclusion thesis

As mentioned above, according to Merleau-Ponty and Legrand, a subject cannot experience his/her own body or body-parts both as-object and as-subject simultaneously. In this section, we argue against this view. We contend that in the case of the four-hand illusion the experience of body-as-object and the experience of body-as-subject can take place concurrently. More precisely, in the synchronous condition of Experiment 3, the *1PP-hands* were simultaneously experienced both as the object of intentionality and as exercising agency. This gave rise to a novel experience in which the experience of body-as-object and the experience of body-as-subject are compatible with each other and do not alternate, such that it presents a serious challenge to the Experiential Exclusion Thesis. Let us elaborate.

It is relatively easy to explain why the 1PP-hands were experienced as-object. In the four-hand illusion, the 1PP-hands were the objects of the participants' vision. The participants' subjective experiences of the 1PP-hands fit Merleau-Ponty's specification of body-as-object that can be observed or handled: “the right hand as an object is … a system of bones, muscles and flesh brought down at a point of space” (1945/1962, P. 105). Using Legrand's words, as the participants watched the 1PP-hands via the HMD, they were *not* absent-as-intentional-object. Rather, the 1PP-hands were “taken *as-intentional-object*, i.e., as the object toward which one's intentional act of consciousness is directed” (Legrand, 2010, p. 187). Thus, during the illusion the 1PP-hands were experienced as-object.

Things are more complicated regarding whether the 1PP-hands were experienced as-subject as well. Three sets of considerations suggest that the answer is yes. First, when the participants experienced the four-hand illusion, they not only felt as if the 1PP-hands were theirs but also felt as if the tactile sensations were located in those hands. More importantly, the participants also felt as if they could control the 1PP-hands. These results fit perfectly Merleau-Ponty's characterization of body-as-subject that “the right hand as it touches … shoots through space like a rocket to reveal the external object in its place” (1945/1962, p. 105). For example, by the synchronous and active finger movement the participants' experiences of the 1PP-hands revealed the surface and the location of the table that they tapped. The 1PP-hands were experienced as-subject because the participants experienced synchronized kinesthetic sense of movement and visual sense of movement in those hands. The participants' subjective experiences of the 1PP-hands fit Legrand's characterizations of body-as-subject as well: “the body as it is acting and perceiving, that is, the body as the point of convergence of action and perception” (2006, p. 108, original emphasis). Legrand endorses Merleau-Ponty's example mentioned above and says: “The paradigmatic example is the experience of the hand-as-touching (vs. the hand-as-touched), which is not an object of experience but is experienced as-subject correlatively with the object touched” (2010, p. 189, original emphasis). Hence, the data collected in the synchronous condition of Experiment 3 strongly suggest that the participants experienced the 1PP-hands as-subject.

Second, the participants' experiences of the 1PP-hands were perfectly consistent with Legrand's other characterizations of the experience of body-as-subject: (1) Legrand characterizes an “experiencing subject” as an “experiencing body” (2010, p. 191) and a “bodily agent” (2007b, p. 503), and says that “the perceiving self corresponds to the body-as-subject” (2010, p. 188). When experiencing synchronous tactile sensations and performed tapping activities, those participants who experienced the four-hand illusion experienced themselves as a perceiving self as well as an experiencing body and a bodily agent. (2) Legrand says: “Experience of the touching hand … corresponds to what I call here pre-reflective bodily consciousness. At this level, the body … is the subject of experience and it is experienced as such” (2007b, p. 499). The participants' experience of the 1PP-hands corresponded to what she calls “pre-reflective bodily consciousness” and hence was an experience of body-as-subject according to Legrand's description here. (3) Legrand also says: “one's body-as-subject-in-the-world is pervasively experienced as it structures any experience, by anchoring it to the spatio-temporal location of the experiencer's body” (2010, p. 190). This was nicely exemplified by the participants' experience of “subjective tactile location”: due to visual manipulation in our experiment, the synchronous tactile stimulations were felt as if they were located on the hands seen from the participants' 1PP.

Third, Kelly (2002) provides a rather different interpretation of Merleau-Ponty's example of touching one's right hand with the left hand. In Kelly's interpretation, “body-as-subject” is specified in terms of “motor intentionality.” The most important feature of motor intentionality, according to Kelly, is that a bodily activity that exhibits motor intentionality “does not have at its heart the kind of autonomous representational content that a subject could have an attitude toward” (2002, p. 390). That is, the distinction between content and attitude does not apply to motor intentional activity such as grasping an object. We would like to make two remarks to suggest that our view with regard to the experience of the 1PP-hands is in fact compatible with Kelly's interpretation.

(1) Consider how Kelly characterizes the minimum requirement for the content/attitude distinction to apply:

“One standard way to characterize a belief state is in terms of a proposition consisting of concepts possessed by the subject enjoying the belief. If Sally believes that the slot is oriented at 45°, for instance, then we may say that Sally possesses the concepts [slot] and [oriented at 45°]. At a minimum this means that she is capable of entertaining at least some other thoughts involving these concepts—thoughts about slots that are not oriented at 45°, for instance, and thoughts about things other than slots that are so oriented. The proposition consisting of the concepts [slot] and [oriented at 45°] is a representation of the way the world is toward which Sally has the attitude of belief (2002, p. 387).”

There are good reasons to consider that the participants who experienced the four-hand illusion did not meet this minimum requirement. The four-hand illusion was a novel illusion first reported by Chen et al. (2018), and the participants were all naïve subjects. This illusion was involuntary and a new experience to the participants. Hence, it is safe to assume that the participants did not possess concepts like [me having four hands], [four-hand body], or [subjective tactile location], etc., to apply those concepts to themselves when the illusion first occurred to them. Thus, there was at least a period of time during which they were not capable of entertaining some other relevant thoughts, and hence they did not have “the kind of autonomous representational content that a subject could have an attitude toward.”

(2) Kelly uses various cases to illustrate his view regarding why the content/attitude distinction does not apply to motor intentional activity. If we consider Kelly's characterizations of these cases, we will see that the 1PP-hand experiences in the four-hand illusion fit those characterizations as well. For example, when discussing grasping activities, Kelly says that “the understanding of the entire object that I have when I am grasping it is not an understanding I can have independent of my bodily activity with respect to it” (2002, p. 385), and that “our bodily understanding is itself a kind of understanding of the object” (2002, p. 386). Similarly, when the participants experienced the illusory senses of ownership and agency in the 1PP-hands, these experiences embodied a kind of understanding that the 1PP-hands were experienced as-subject. The experiences of the 1PP-hands as-subject were not independent of the participants' sense of ownership and the sense of agency. We are not suggesting that our view and goal are the same as Kelly's. While Kelly's discussions are mainly about bodily understanding of external objects, we focus on the subjective experience of the 1PP-hands involved in the four-hand illusion. Still, our view is compatible with Kelly's interpretations.

When commenting on the case of unreflectively opening the door, Kelly says that “I sometimes seem to be able to remember, for instance, reaching out to grasp the doorknob, even if I wasn't aware of doing it when I actually performed the activity … motor intentionality is like this even for normal subjects, that it essentially discloses the world to us, in other words, but cannot be captured in the process of doing so” (2002, p. 389). Kelly also cites the report by a patient named Schneider mentioned by Merleau-Ponty: “I experience the movements as being a result of the situation, of the sequence of events themselves … I am scarcely aware of any voluntary initiative. … It all happens independently of me” (2002, p. 390). Now consider our case. The study by Chen et al. (2018) showed that the four-hand illusion is a genuine bodily illusion. That means, it is a subjective experience induced by the experimental set-up, not a conceptual judgment. The participants were aware of their finger movement and what they saw via the HMD, but they had no idea why and how the illusion occurred to them, including the illusory senses of ownership and agency of the 1PP-hands. More importantly, the onset of the illusion was involuntary. They were, so to speak, “scarcely aware of any voluntary initiative,” and they felt that “It all happens independently of me.” In this regard, we think that their experiences fit Kelly's characterizations of motor intentionality.

Together, the above considerations strongly support that, in the four-hand illusion, the 1PP-hands were experienced as-subject. It is crucial to notice that the 1PP-hands can be experienced as-subject only because the participants continued to watch them. The idea of alteration embedded in the Experiential Exclusion Thesis fails to apply here. To see this point, consider the kinesthetic sense of movement and the visual sense of movement involved in Experiment 3. For those participants who experienced the four-hand illusion, in addition to receiving synchronous tactile stimulations, their experiences of the 1PP-hands had the following features. On the one hand, the participants experienced active finger movement via kinesthesis and proprioception with regard to their real hands. On the other hand, they also saw the active finger movement with regard to the hands viewed from the adopted 1PP (1PP-seen movement). The kinesthetic sense of movement was partially captured by the vision of the 1PP-hand movement^10^. Both were experienced concurrently and were integrated with each other. If the kinesthetic sense of movement and the visual sense of movement were not linked together in this way, the participants would not feel as if the 1PP-hands were theirs and as if they could control them. That means, the participants would not experience the 1PP-hands as-subject *unless* the 1PP-hands were also experienced as the objects of vision. This shows that the integrated experience of the 1PP-hands cannot be explained away by the alteration account. The synchronous condition of Experiment 3 created a situation where one can simultaneously experience the same body-parts, i.e., the 1PP-hands, *both* as-subject and as-object. In this case, the two modes of experience are not incompatible. Hence, the Experiential Exclusion Thesis was violated by the experience of the 1PP-hands in the four-hand illusion. *Pace* Merleau-Ponty, it is *not* always true that “In so far as it sees or touches the world, my body can therefore be neither seen nor touched” (1945/1962, p. 105). We will discuss this further in the next section.

Our claim is that in the case of the four-hand illusion, the sense of body ownership, subjective tactile location and the sense of agency in the 1PP-hands jointly constitute a counter-example against the Experiential Exclusion Thesis^11^. There are two important provisos: first, we are not making a broader claim that any case will be jointly sufficient for the feel of body-as-subject as long as these three components are involved. We only claim that for those participants who experienced the four-hand illusion, their experiences of the 1PP-hands constitute an experience of body-as-subject. In order to challenge the Experiential Exclusion Thesis, what we need is one single counter-example, not the broader claim. Second, our view is that in order for the sense of body ownership, subjective tactile location and the sense of agency to constitute a simultaneous experience of body-as-object and body-as-subject, these three components must be linked in a specific way: not only they happen concurrently, but also they must be integrated in the way arranged by our experimental set-up, such that the 1PP-hands would not be experienced as-subject unless they were also experienced as the objects of vision at the same time.

## Objections and responses

In this section, we consider some potential objections: first, a defender of the Experiential Exclusion Thesis might appeal to Merleau-Ponty's case of “double sensations” to reply to our argument. As described in the Introduction, the idea of this case is that, although both of my hands are physically pressing each other and being pressed at the same time, the experiences of pressing and being pressed are still experientially incompatible such that their roles alternate between the two hands. The defender might apply this idea to the four-hand illusion and maintain the following stance: although the participants experienced an illusory sense of agency in the 1PP-hands and at the same time watched them from the adopted 1PP, *at the experiential level* the sense of body-as-subject and the sense of body-as-object remained incompatible and alternated in the participants' subjective experiences. If so, since they were not experienced simultaneously, the Experiential Exclusion Thesis still holds.

We disagree. Without empirical support, this defense would be merely based on a conceptual stipulation. We think that the visual and agentive experiences of the 1PP-hands did not alternate in the way suggested by this defense. Not only that they took place concurrently, but also that they were integrated experientially so as to contribute to the four-hand illusion. We can agree that mere temporal simultaneity would not rule out the alteration account. Our view is that it is the integration of those experiences that hinders the idea of experiential incompatibility in that account. The above defense fails to recognize a key difference between Merleau-Ponty's case and ours. That is, it underestimates the essential role that vision plays in the four-hand illusion. As in many full-body illusions (Ehrsson, 2007; Lenggenhager et al., 2007; Petkova and Ehrsson, 2008), the visual perspective of the participants was manipulated in certain ways. None of the illusory effects, including the illusory sense of ownership, agency, etc., could be induced without the visual manipulation. In our experiment, the methodological integration of visual manipulation, tactile stimulations and finger movements bound the sense of body-as-object and the sense of body-as-subject together in the experience of the 1PP-hands. As we argued above, the participants would not experience the 1PP-hands as-subject unless the same hands were also experienced as the objects of vision^12^. This makes it implausible to interpret the participants' experiences as alternating between as-subject and as-object. In contrast, such a methodological integration did not figure in Merleau-Ponty's considerations. Vision does not play any particular role either in his example of touching one's right hand with the left hand or in his case of “double sensations.” Therefore, the above defense does not really show that the case of the 1PP-hands can be accommodated by experiential incompatibility, and hence does not save the Experiential Exclusion Thesis from our argument based on the four-hand illusion.

Second, some philosophers might wonder how reliably our questionnaire data and statistics can tell us about the participants' subjective experiences. This is a methodological issue regarding the relationship between the cognitive or reflective judgments measured by the questionnaires and the subjective or pre-reflective experiences of the illusion. Here are our responses: (1) although there were no necessary connections between cognitive judgments and subjective experiences, all of the participants in our experiments were healthy subjects. It does not seem plausible to insist that there must be a fundamental gap between their judgments in the questionnaires and their subjective experiences. (2) As Zahavi says, “Reflection is constrained by what is pre-reflectively lived through. It is answerable to experiential facts and is not constitutively self-fulfilling. To deny that the reflective self-ascription of beliefs is based on any experiential evidence whatsoever is implausible” (Zahavi, 2014, p. 36; cf. also his 2005, p. 95–96). We welcome this remark, as it suggests that the participants' cognitive judgments were constrained by, and hence could reveal, their subjective experiences of the 1PP-hands. (3) Our questionnaire results were supported by SCR measurements (Chen et al., 2018). It is widely recognized that SCR cannot be voluntarily affected by the participants, hence can provide objective evidence for questionnaires.

Third, consider Legrand's other characterization of body-as-subject in Merleau-Ponty's example: “the body is experienced-as-subject correlatively to things in the world perceived-as-object. The paradigmatic example is the experience of the hand-as-touching (vs. the hand-as-touched), which is not an object of experience but is experienced as-subject correlatively with the object touched” (Legrand, 2010, p. 189). Here, Legrand suggests that there exists a correlative relationship between the experience of the hand-as-touching and the experience of the hand-as-touched. Based on this characterization, one might raise the following criticism: since the 1PP-hands were actually the experimenter's hands, not the participants' hands, the participants did not really experience an object through the 1PP-hands. Thus, there is no correlative relationship to be had in our case.

We do not think so. When Legrand talks about the correlative relationship in Merleau-Ponty's example, the phrase “things in the world perceived-as-object” refers to the right hand-as-touched. Now, as stated above, we think that the participants' kinesthetic sense of movement was partially captured by the vision of the 1PP-hand movement, such that when they tapped on the table with their own index fingers they felt as if they were tapping with the 1PP-hands. Although the 1PP-hands were actually the experimenter's hands, that did not prevent the participants from experiencing those hands as if they owned and could control them. The illusory senses of agency and ownership of the 1PP-hands were generated by the integration of the kinesthetic and visual sense of finger movement, as well as feeling external tactile stimulations and watching all four hands being touched synchronously. So the experience of the 1PP-hands was an integrated experience, in which the 1PP-hands were experienced as-subject correlatively to the same hands experienced as-object in that they were at the same time experienced as the objects of vision. In this sense, the 1PP-hands were experienced-as-subject “correlatively to things in the world perceived-as-object,” and the former would not occur without the latter.

Finally, one might object that, even if our counter-example succeeds in refuting the Experiential Exclusion Thesis, it could turn out to be a trivial one. Suppose I simply tap on a table with both hands and look at them. I could then easily experience ownership and agency over my own hands, and at the same time experience the same hands as intentional objects of visual awareness. Why not take this to serve as a much simpler counter-example? If so, the intricate setup of our experiment and the discussions above about the 1PP-hand experiences all begin to look redundant.

For the sake of discussion, let us call the situation described above “the ordinary case.” In the ordinary case, the subject does not wear an HMD and there are no visual and tactile manipulations. The finger movement of the one's own hands and the visual experience of the same hands take place concurrently. If we consider Merleau-Ponty's example of touching one's right hand with the left hand, we can see that it is a type of ordinary case as well: “I can, with my left hand, feel my right hand as it touches an object.” In this example, the tactile experience on the right hand and the movement of the same hand take place at the same time. Merleau-Ponty says that “In so far as it sees or touches the world, my body can therefore be neither seen nor touched.” He could easily apply this claim to the ordinary case and say: in so far as my hands tap on the table, they can be neither seen nor touched. From Merleau-Ponty's standpoint, the ordinary case can be characterized in terms of experiential incompatibility of body-as-object and body-as-subject. These considerations indicate that, for the defender of the Experiential Exclusion Thesis, it would not count as a counter-example against the thesis if two experiences merely take place simultaneously on the same body or body-parts. In this paper, we only aim to show that there exist at least one genuine counter-example against the Experiential Exclusion Thesis. So for our purpose, we can grant that Merleau-Ponty's view works for the ordinary case.

The main difference between the ordinary case and the subjective experience of the 1PP-hands in the four-hand illusion (call this “the experimental case”) is the following: in the ordinary case, my finger movement and visual experience of my own hands are not integrated as in the experimental case. They only happen concurrently, but each can occur without the other: I can tap my own hands against the table without watching them and can look down at my own hands without tapping them. The tapping experience and the visual experience are separable. The experimental case was very different in this regard. As suggested above, the participants' finger movement and their vision of the 1PP-hand movement not only took place synchronously but also integrated such that their experiences of ownership, agency and subjective tactile location of the 1PP-hands would not occur unless the same hands were also the objects of their vision. In the experimental case, Merleau-Ponty's claim fails to apply: the 1PP-hands cannot be experienced as mine and as touching the world without being visible. On the contrary, the visual experience of the 1PP-hands was part of the reason why they were experienced as mine and as touching the world. The alteration account does not work here. Hence the experimental case cannot be replaced by the ordinary one.

There is another important difference between the ordinary and the experimental cases. As suggested above, the content/attitude distinction does not apply to the experimental case. In contrast, this distinction seems to apply to the ordinary case. Unlike the four-hand illusion, the tapping activity in the ordinary case is not a novel experience. It is safe to assume that the subject already possesses concepts like [my hands], [index fingers], and [looking down], etc., and is capable of entertaining some other thoughts involving these concepts, for example, thoughts about tapping with toes rather than index fingers and thoughts about looking down at my belly rather than hands, etc. Thus, the representational content involved in the ordinary case is of the kind that “a subject could have an attitude toward” (Kelly, 2002, p. 390).

Final remarks: to our best knowledge, the four-hand illusion provides the first counter-example in the literature that challenges Merleau-Ponty's view about the body-as-object and body-as-subject. Once this is established, it opens up the possibility that different empirical studies of bodily illusions might supply other counter-examples against the Experiential Exclusion Thesis as well. Given the vast varieties of experimental set-ups that have been used in the research of bodily illusions, any other potential counter-examples would have to be examined case by case. Although a full discussion on this issue will exceed the scope of this paper, we would like to make the following observations.

First, not all bodily illusions will challenge the Experiential Exclusion Thesis. In the standard rubber-hand illusion (RHI) (Botvinick and Cohen, 1998; Tsakiris and Haggard, 2005), the subjects watched a fake hand being stroked synchronously with their own unseen hand. Since the fake hand was passively touched and was an object of vision, it was only an experience of body-as-object. As variants of RHI, the participants in Ehrsson (2009) watched two fake hands stroked synchronously with respect to their own hidden hand, and the participants in Guterstam et al. (2011) saw their real hand adjacent to a rubber hand being stroked synchronously with each other. The subjects in both of these two studies felt as if they have two right hands. Again, since the two right hands were viewed and touched passively, they were objects of vision and touch. Hence, these variants of RHI involve only experiences of body-as-object. Similarly, in the study of full-body illusion by Lenggenhager et al. (2007), the participants watched their virtual body in the front and passively receive tactile stimulations on the back. As Legrand characterizes it, “in ‘Lenggenhagerian' OBEs (Lenggenhager et al., 2007) one experiences one's body-as-object as being in a location where the biological body is not (the same goes for the RHI)” (2010, p. 193). We agree. Thus, the Experiential Exclusion Thesis remains intact with regard to these studies.

Second, more promising cases can be found in other variants of RHI experiments where agency is involved. Kalckert and Ehrsson (2012) report that, in the active congruent condition, the sense of agency was stronger when the rubber hand was experienced as a part of one's body. Dummer et al. (2009) also suggest that the sense of body ownership was stronger if RHI was induced by active movement. In the study by Riemer et al. (2013), the proprioceptive drift was stronger in actively moving RHI than that in RHI without movement. Two points together suggest that these cases are potential threats to Merleau-Ponty's view: (i) Both the sense of body ownership and the sense of agency are positively involved in Merleau-Ponty's example of touching one's right hand with the left hand. (ii) Just like our counter-example, in these cases body ownership, active movement and visual experience of the fake hand were integrated such that the illusory sense of ownership and agency would not be induced without the participants looking at the rubber hand.

However, there is an important proviso. The relationship between body ownership and agency can vary considerably, depending on experimental set-up. For example, in other conditions of the same study, Kalckert and Ehrsson (2012, p. 1, 9–12) found that “passive movements abolished the sense of agency but left ownership intact, and incongruent positioning of the model hand diminished ownership but did not eliminate agency.” Based on these findings, Kalckert and Ehrsson suggest that the sense of body ownership and the sense of agency can be dissociated. Also, Walsh et al. (2011, p. 3019) report that “active congruent movements (i.e., voluntary movements) produced an illusion that was the same or weaker than that produced by passive congruent movements.” Unlike Merleau-Ponty's example, in these cases body ownership and agency did not both contribute positively in the experience of RHI. The sense of body ownership, the sense of agency and visual experience of the fake hand in these cases were not as integrated as in our counter-example. It is not obvious whether they would threaten Merleau-Ponty's view. Hence, we think that probably not all bodily illusions that involve hand movement would undermine the Experiential Exclusion Thesis.

To conclude: on the one hand, we do not claim that the four-hand illusion is the only case in which the same body-parts can be simultaneously experienced both as-object and as-subject. We only claim that our counter-example is the first one reported in the literature. On the other hand, it is not the case that Merleau-Ponty's view will in general be undermined by any bodily illusions. Between these two ends, we suspect that the Experiential Exclusion Thesis could be challenged by some other experimental cases. Given the multifarious phenomena reported in this research area, it is possible that potential counter-examples would come in degrees. The case of the four-hand illusion constitutes a strong counter-example because it integrates body ownership, agency, subjective tactile location and vision that are associated with experiences of both body-as-object and body-as-subject, and it rules out the view that the experiences of the subjects are switching between the two modes of experience. This case provides a useful basis for comparing with other potential ones. Further investigations will be required to verify whether and how exactly other experimental cases might have impact on the Experiential Exclusion Thesis.

## Conclusion

It is possible that body-as-object and body-as-subject can be experienced simultaneously. For the record, we do not think that the distinction between body-as-subject and body-as-object should be annihilated. We only argue that this distinction is not as rigid as suggested by the phenomenologists. The experience of body-as-object and the experience of body-as-subject are *not* mutually exclusive. A positive implication is that, since the distinction is not rigid, our view opens up the possibility that neuroscientific research of body-as-object, e.g., studies on body ownership, could shed light on body-as-subject. On the one hand, as one of us has suggested elsewhere, one should not take it for granted that a neuroscientific explanation of body-as-object can automatically apply to body-as-subject (Liang, 2016). On the other hand, as shown in the case of the four-hand illusion, these two types of self-experiences are not totally unrelated, either. Therefore, a future task would be to conduct interdisciplinary research to further investigate the relationship between the sense of body-as-object and the sense of body-as-subject.

## Author contributions

CL wrote this manuscript. Y-TL, W-YC and H-CH helped refining early versions of the manuscript.

### Conflict of interest statement

The authors declare that the research was conducted in the absence of any commercial or financial relationships that could be construed as a potential conflict of interest.

## Footnotes

^1^When Legrand specifies body-as-subject, she takes Merleau-Ponty's case cited above as an example, which is a case of body-part. She says that “Experience of the touching hand … corresponds to what I call here pre-reflective bodily consciousness. At this level, the body … is the subject of experience and it is experienced as such” (2007b, p. 499). We agree with her on this point. Our position differs from Legrand's when it comes to the Experiential Exclusion Thesis. See below.

^2^For example, when I look at some flowers in front of me, I do this with my eyes, but I don't thereby see my own eyes. If I want to bring some of the flowers with me to the school, I would take hold of them and put them in my bike basket. But when I move, my eyes (as well as the rest of my body) spontaneously move with me, and I don't need to do anything to make this happen. I don't need to look for my eyes, as they are already and always with me.

^3^Also, cf. the discussion on non-observational bodily awareness by Kühle (2017).

^4^Totally we conducted six experiments and recruited 150 subjects. For their full descriptions and results, cf. Chen et al. (2018). Here, we argue for the philosophical implication of the four-hand illusion.

^5^How is “body-as-subject” discussed here related to “self-as-subject” in the philosophical literature? Our view is that although body-as-subject and self-as-subject are related, the exact relationship between them is complicated. A full treatment of this issue will go beyond the scope of this paper, but here are what we think: most discussions on self-as-subject center on the so-called “immunity principle” (immunity to error through misidentification relative to the first-person pronoun, hereafter, *IEM*) and regard it as a purely conceptual or semantic issue (Shoemaker, 1968; Pryor, 1999; Coliva, 2006; Prosser and Recanati, 2012). They concern whether sentences using the first-person pronoun “I” or cognitive states involving first-person concept “I” enjoy IEM. In contrast, Merleau-Ponty's remarks on body-as-subject focus on bodily experiences. He is more concerned with how the experiences of one's body differ from the experiences of other external objects. Legrand's discussions (2006, 2007a) can bring body-as-subject and self-as-subject together because she thinks that (i) the self is essentially bodily, and (ii) IEM is correct (cf. also Gallagher, 2005, 2012; Zahavi, 2005, 2014). So for Legrand, “body-as-subject” and “self-as-subject” could mean the same thing, because self-as-subject is to be construed as bodily self-as-subject and “the body … is the subject of experience and it is experienced as such” (2007b, p. 499). Our view is different: we subscribe to (i) but not (ii). We think that IEM, at least some version of it, faces serious counter-examples when it comes to bodily experiences such as tactile sensations (cf. Liang et al., 2015; Liang, 2016). Since we accept that the self is essentially bodily, we can agree that in many cases the distinction between body-as-subject and body-as-object overlaps with the distinction between self-as-subject and self-as-object. Many daily examples that can be explained by the former distinction can also be explained by the latter. In this sense, body-as-subject and self-as-subject are closely related. However, we are not prepared to say that body-as-subject and self-as-subject are the same. The reason is that while many philosophers regard IEM as the core of “self-as-subject,” it is not obvious whether IEM is essential for “body-as-subject” at all. Merleau-Ponty does not (nor does he need to) rely on something like IEM to defend what we call the Experiential Exclusion Thesis. Given these considerations, in order to determine the exact relationship between body-as-subject and self-as-subject, several issues would need to be dealt with, including the following: how, if at all, does IEM bear on the notion of body-as-subject? Must the idea of self-as-subject be explicated in terms of IEM? What is the relationship between IEM and the Experiential Exclusion Thesis? These issues exceed the scope of this paper. In this paper, we focus on body-as-subject, the Experiential Exclusion Thesis, and the four-hand illusion.

^6^When attaching color tags, an irrelevant landscape image was shown on the HMD so that the subject would not know whether the tags on his/her own hands were red or blue. After this was done, the screen was switched back to the images taken by the stereo camera. The descriptions in this section are adopted from Chen et al. (2018).

^7^Experiments 4 ~ 6 were three other active four-hand conditions that served as control groups, cf. Table 3 in Chen et al. (2018).

^8^This finding was consistent with the “double body illusion” reported by Heydrich et al. (2013).

^9^For more details about the experimental data and statistical analyses, cf. Chen et al. (2018).

^10^The reason why the visual capture here was only partial was because in the synchronous condition the experience of the 1PP-hands did not hinder the ownership and agency of the 3PP-hands (cf. Chen et al., 2018).

^11^Here, “subjective tactile location” refers to the phenomenon that, due to visual manipulation in our experiment, the synchronous tactile stimulations were felt as if they were located on the hands seen from the participants' 1PP.

^12^The role of vision is crucial in our counter-example against the Experiential Exclusion Thesis. However, we do not think that vision by itself can explain why, in the four-hand illusion, the 1PP-hands were experienced both as-object and as-subject simultaneously. In order for the illusion to occur, the sense of body ownership, the subjective tactile location, and the sense of agency in the 1PP-hands must not only occur together, but also must be integrated in certain ways, such that the 1PP-hands would not be experienced as-subject unless they were also experienced as the objects of vision at the same time. Hence, we think that vision alone would not suffice for a body or body part to be experienced simultaneously both as-subject and as-object. Other factors must come into play as well.

## References

[B1] Botvinick M. Cohen J. (1998). Rubber hands ‘feel' touch that eyes see. Nature 391:756. 10.1038/35784 9486643

[B2] Chen W. Y. Huang H. C. Lee Y. T. Liang C. (2018). Body ownership and the four-hand illusion. Sci. Rep. 8:2153. 10.1038/s41598-018-19662-x 29391505 PMC5794744

[B3] Coliva A. (2006). Error through misidentification: some varieties. J. Philos. 103, 403–425. 10.5840/jphil2006103824

[B4] Dummer T. Picot-Annand A. Neal T. Moore C. (2009). Movement and the rubber hand illusion. Perception 38, 271–280. 10.1068/p5921 19400435

[B5] Ehrsson H. H. (2007). The experimental induction of out-of-body experiences. Science 317:1048. 10.1126/science.1142175 17717177

[B6] Ehrsson H. H. (2009). How many arms make a pair? Perceptual illusion of having an additional limb. Perception 38, 310–312. 10.1068/p6304 19400438

[B7] Gallagher S. (2005). How the Body Shapes the Mind. Oxford: Clarendon Press.

[B8] Gallagher S. (2012). First-person perspective and immunity to error through misidentification, in Consciousness and Subjectivity, eds Miguens S. Preyer G. (Frankfurt: Philosophical Analysis Ontos Publisher), 187–214. 10.1515/9783110325843.245

[B9] Guterstam A. Petkova V. I. Ehrsson H. H. (2011). The illusion of owning a third arm. PLoS ONE 6:e17208. 10.1371/journal.pone.0017208 21383847 PMC3044173

[B10] Heydrich L. Dodds T. J. Aspell J. E. Herbelin B. Bülthoff H. H. Mohler B. J.. (2013). Visual capture and the experience of having two bodies–evidence from two different virtual reality techniques. Front. Psychol. 4:946. 10.3389/fpsyg.2013.00946 24385970 PMC3866547

[B11] Kalckert A. Ehrsson H. H. (2012). Moving a rubber hand that feels like your own: a dissociation of ownership and agency. Front. Hum. Neurosci. 6:40. 10.3389/fnhum.2012.00040 22435056 PMC3303087

[B12] Kelly S. D. (2002). Merleau–Ponty on the body. Ratio 15, 376–391. 10.1111/1467-9329.00198

[B13] Kühle L. (2017). The missing pieces in the scientific study of bodily awareness. Philos. Psychol. 30, 571–593. 10.1080/09515089.2017.1311999

[B14] Legrand D. (2006). The bodily self: the sensori-motor roots of pre-reflective self-consciousness. Phenomenol. Cogn. Sci. 5, 89–118. 10.1007/s11097-005-9015-6

[B15] Legrand D. (2007a). Pre-reflective self-as-subject from experiential and empirical perspectives. Conscious. Cogn. 16, 583–599. 10.1016/j.concog.2007.04.002 17533140

[B16] Legrand D. (2007b). Pre-reflective self-consciousness: on being bodily in the world. Janus Head 9, 493–519.

[B17] Legrand D. (2010). Myself with no body? body, bodily-consciousness and self-consciousness, in Handbook of Phenomenology and Cognitive Science (London: Springer), 180–200. 10.1007/978-90-481-2646-0_10

[B18] Lenggenhager B. Tadi T. Metzinger T. Blanke O. (2007). Video ergo sum: manipulating bodily self-consciousness. Science 317, 1096–1099. 10.1126/science.1143439 17717189

[B19] Liang C. (2016). Self-as-subject and experiential ownership, in Open MIND: Philosophy and the Mind Sciences in the 21st Century, Vol. 2, eds Metzinger T. Windt J. M. (London: The MIT Press), 957–975.

[B20] Liang C. Chang S. Y. Chen W. Y. Huang H. C. Lee Y. T. (2015). Body ownership and experiential ownership in the self-touching illusion. Front. Psychol. 5:1591. 10.3389/fpsyg.2014.01591 25774138 PMC4344111

[B21] Merleau-Ponty M. (1945/1962). Phenomenology of Perception. Transl. by C. Smith. London: Routledge.

[B22] Petkova V. I. Ehrsson H. H. (2008). If I were you: perceptual illusion of body swapping. PLoS ONE 3:e3832. 10.1371/journal.pone.0003832 19050755 PMC2585011

[B23] Prosser S. Recanati F. (Eds). (2012). Immunity to Error Through Misidentification: New Essays. Cambridge: Cambridge University Press.

[B24] Pryor J. (1999). Immunity to error through misidentification. Philos. Top. 26, 271–304.

[B25] Riemer M. Kleinböhl D. Hölzl R. Trojanc J. (2013). Action and perception in the rubber hand illusion. Exp. Brain Res. 229, 383–393. 10.1007/s00221-012-3374-3 23307154

[B26] Shoemaker S. (1968). Self-reference and self-awareness. J. Philos. 65, 555–567. 10.2307/2024121

[B27] Tsakiris M. Haggard P. (2005). The rubber hand illusion revisited: visuotactile integration and self-attribution. J. Exp. Psychol. Hum. Percept. Perform. 31, 80–91. 10.1037/0096-1523.31.1.80 15709864

[B28] Walsh L. D. Moseley G. L. Taylor J. L. Gandevia S. C. (2011). Proprioceptive signals contribute to the sense of body ownership. J. Physiol. 589, 3009–3021. 10.1113/jphysiol.2011.204941 21521765 PMC3139083

[B29] Zahavi D. (2005). Subjectivity and Selfhood: Investigating the First-Person Perspective. Cambridge: MIT press.

[B30] Zahavi D. (2014). Self and Other: Exploring Subjectivity, Empathy, and Shame. Oxford: Oxford University Press.

